# Microwave ablation is as effective as radiofrequency ablation for very-early-stage hepatocellular carcinoma

**DOI:** 10.1186/s40880-017-0183-x

**Published:** 2017-01-19

**Authors:** Yun Xu, Qiang Shen, Neng Wang, Pan-Pan Wu, Bin Huang, Ming Kuang, Guo-Jun Qian

**Affiliations:** 1 Department of Minimal Invasion Therapy, Eastern Hepatobiliary Surgery Hospital, The Second Military Medical University of Chinese PLA, 225# Changhai Rd, Shanghai, 200438 P. R. China; 2 Department of Radiology, Eastern Hepatobiliary Surgery Hospital, The Second Military Medical University of Chinese PLA, Shanghai, 200438 P. R. China; 3 Division of Interventional Ultrasound, Department Hepatobiliary Surgery, The First Affiliated Hospital of Sun Yat-sen University, 58# Zhongshan Road 2nd, Guangzhou, 510080 Guangdong P. R. China

**Keywords:** Microwave ablation, Radiofrequency ablation, Hepatocellular carcinoma

## Abstract

**Background:**

Percutaneous radiofrequency ablation (RFA) is a first-line treatment for very-early-stage hepatocellular carcinoma (HCC), whereas the efficacy of percutaneous microwave ablation (MWA) for very-early-stage HCC remains unclear. The purpose of this study was to clarify this issue by comparing the safety and efficacy of percutaneous MWA with percutaneous RFA in treating very-early-stage HCC.

**Methods:**

Clinical data of 460 patients who were diagnosed with very-early-stage HCC and treated with percutaneous MWA or RFA between January 2007 and July 2012 at the Eastern Hepatobiliary Surgery Hospital, The Second Military Medical University, in Shanghai, China were retrospectively analyzed. Of these 460 patients, 159 received RFA, 301 received MWA. Overall survival (OS), recurrence-free survival (RFS), local tumor progression (LTP), complete ablation, and complication occurrence rates were compared between the two groups, and the prognostic factors associated with survival were analyzed.

**Results:**

No significant differences were observed between the two groups in terms of the 1-, 3-, or 5-year OS rates (99.3%, 90.4%, and 78.3% for MWA vs. 98.7%, 86.8%, and 73.3% for RFA, respectively; *P* = 0.331). Furthermore, no significant differences were observed between the two groups in terms of the corresponding RFS rates (94.4%, 71.8%, and 46.9% for MWA vs. 89.9%, 67.3%, and 54.9% for RFA, respectively; *P* = 0.309), the LTP rates (9.6% vs. 10.1%, *P* = 0.883), the complete ablation rates (98.3% vs. 98.1%, *P* = 0.860), or the occurrence rates of major complications (0.7% vs. 0.6%, *P* = 0.691). By multivariate analysis, LTP, antiviral therapy, and treatment of recurrence were independent risk factors for OS (*P* < 0.001), and the alpha-fetoprotein level was an independent prognostic factor for RFS (*P* = 0.002).

**Conclusions:**

MWA is as safe and effective as RFA in treating very-early-stage HCC, supporting MWA as a first-line treatment option for this disease.

## Background

Hepatocellular carcinoma (HCC) is the third most common malignancy worldwide; in China, it results in the second highest cancer-related mortality [1]. According to the Barcelona clinic liver cancer (BCLC) management guidelines [2], early-stage HCC (defined as a single HCC lesion ≤5 cm in diameter or three nodules ≤3 cm in diameters) can be potentially cured by liver transplantation, hepatic resection, or thermal ablation. Liver transplantation carries the greatest benefits by replacing the cancerous liver that often results from cirrhotic alterations; however, a shortage of donor livers limits its wide application [3]. Currently, hepatic resection represents the primary treatment option for early-stage HCC.

Thermal ablation, including radiofrequency ablation (RFA) and microwave ablation (MWA), is initially primarily selected for HCC patients who are unsuitable for liver transplantation or hepatic resection [4]. However, during the last two decades, the application of thermal ablation has widened and emerged as an additional first-choice treatment option for early-stage HCC [5–9]. A randomized controlled trial [5] and a cohort of clinical trials [6–8] showed that RFA can result in similar treatment outcomes to those resulted in by hepatic resection. Meanwhile, MWA is increasingly being used to treat HCC. Our previous report suggested that, in treating early-stage HCC, MWA can be as effective as RFA [9]. After extensive studies and clinical practice, thermal ablation has been recommended in the Eastern Hepatobiliary Surgery Hospital for more than 10 years as the first-choice treatment option.

In the clinic, an initial solitary HCC of 2 cm or smaller is referred to as very-early-stage HCC [2, 10]. With the widening applications of radiologic technology for HCC screening, a growing number of HCC patients are now diagnosed at the very early stage. Previous studies showed that 70% of patients with very-early-stage HCC treated with thermal ablation or hepatic resection can achieve 5-year survival [11–17]. Several groups compared the treatment outcomes of RFA and hepatic resection and suggested that RFA can be considered a first-choice treatment option, even if the HCC is resectable [12]. However, the efficacy of MWA for very-early-stage HCC remains unclear. To clarify this issue, we conducted a retrospective study of the effects of MWA versus RFA in treating very-early-stage HCC. Overall survival (OS), recurrence-free survival (RFS), local tumor progression (LTP), complete ablation, and complication occurrence rates were compared between the two groups. Prognostic factors associated with survival were also analyzed.

## Patients and methods

### Ethics statement

All examinations and treatments were conducted at the Eastern Hepatobiliary Surgery Hospital, The Second Military Medical University, in Shanghai, China and were in accordance with the Declaration of Helsinki. This study was approved by the Ethics Committee of the Eastern Hepatobiliary Surgery Hospital. Written informed consent was obtained from all patients included in this study.

### Patients

Between January 2007 and July 2012, 7569 patients with HCC were admitted to the Department of Minimally Invasive Therapy. All very-early-stage HCC patients during the same period who met the following criteria were included in this retrospective study: (a) initial solitary HCC nodule of 2 cm or smaller in size; (b) liver cirrhosis class A or B (according to the Child–Pugh staging system), prothrombin activity greater than 50%, and platelet (PLT) count greater than 50 × 10^9^/L; (c) absence of extrahepatic or vascular metastasis; and (d) thermal ablation as their first-choice treatment.

Tumor size was evaluated by computed tomography (CT) and magnetic resonance imaging (MRI). Because of possible complications arising from seeding cancer cells [18], a percutaneous liver biopsy is not suggested in the Eastern Hepatobiliary Surgery Hospital. The diagnosis of HCC followed the criteria established by the American Association for the Study of Liver Diseases: hepatic lesion mass of 2 cm or smaller as detected with four-phase CT and MRI with intense arterial uptake followed by “washout” of the contrast agent in the portal and delayed phases [3]. Unfavorable tumor locations were defined as nodules located 5 mm or closer to critical structures, including the gallbladder, gastrointestinal tract, hilum, pericardium, diaphragm, and major vessels [19]. Major vessels were defined as the first or second branch of the portal vein, the inferior vena cava, and the main hepatic veins [19]. The presence of “Heredity” refers to the status that any immediate relatives within three generations suffered from HCC.

Individual cases were examined and discussed by our multidisciplinary team, involving hepatologists, interventional radiologists, and surgeons. In this cohort, patients selected underwent either the MWA or RFA procedure if the tumors were not adjacent to major vessels. However, to lower susceptibility to the heat sink effect, MWA was recommended for tumors adjacent to major vessels.

### Ablation procedure

#### MWA device

A FORSEATM MW delivery system (Qinghai Microwave Electronic Institute, Nanjing, Jiangsu, China) was used. This system is composed of an MTC-3 microwave generator with a frequency of 2450 MHz and a power output of 1–100 W, a flexible low-loss coaxial cable, and a 14-gauge cooled shaft antenna. The antenna consists of one 18-cm-long shaft coated with Teflon to prevent tissue adhesion and a 3-cm-long exposed antenna at its terminus with a 1.5-cm-long active tip coated with polytetrafluoroethylene.

#### RFA device

A Cool-tip™ RFA system (Valley Lab, Boulder, CO, USA) was used. This system is composed of a radiofrequency generator with a maximum power output of 200 W, as well as a 17-gauge and 18-cm-long internally cooled needle electrode. A 2-cm-long activating tip electrode was used.

After local anesthesia at the puncture site, under the guidance of real-time ultrasound, the antenna or electrode was percutaneously probed into the tumors, with the tip placed in the deepest part of the nodule. A RFA was applied for 10–12 min. For all patients, the application of MWA at 80–100 W was performed in automatic mode for 3–5 min. A safety margin of more than 1.0 cm was employed for all thermal ablation-treated tumors. At the end of ablation, the puncture tract was coagulated to prevent potential bleeding or tumor seeding.

### Efficacy evaluation and follow-up

**Fig. 1:**
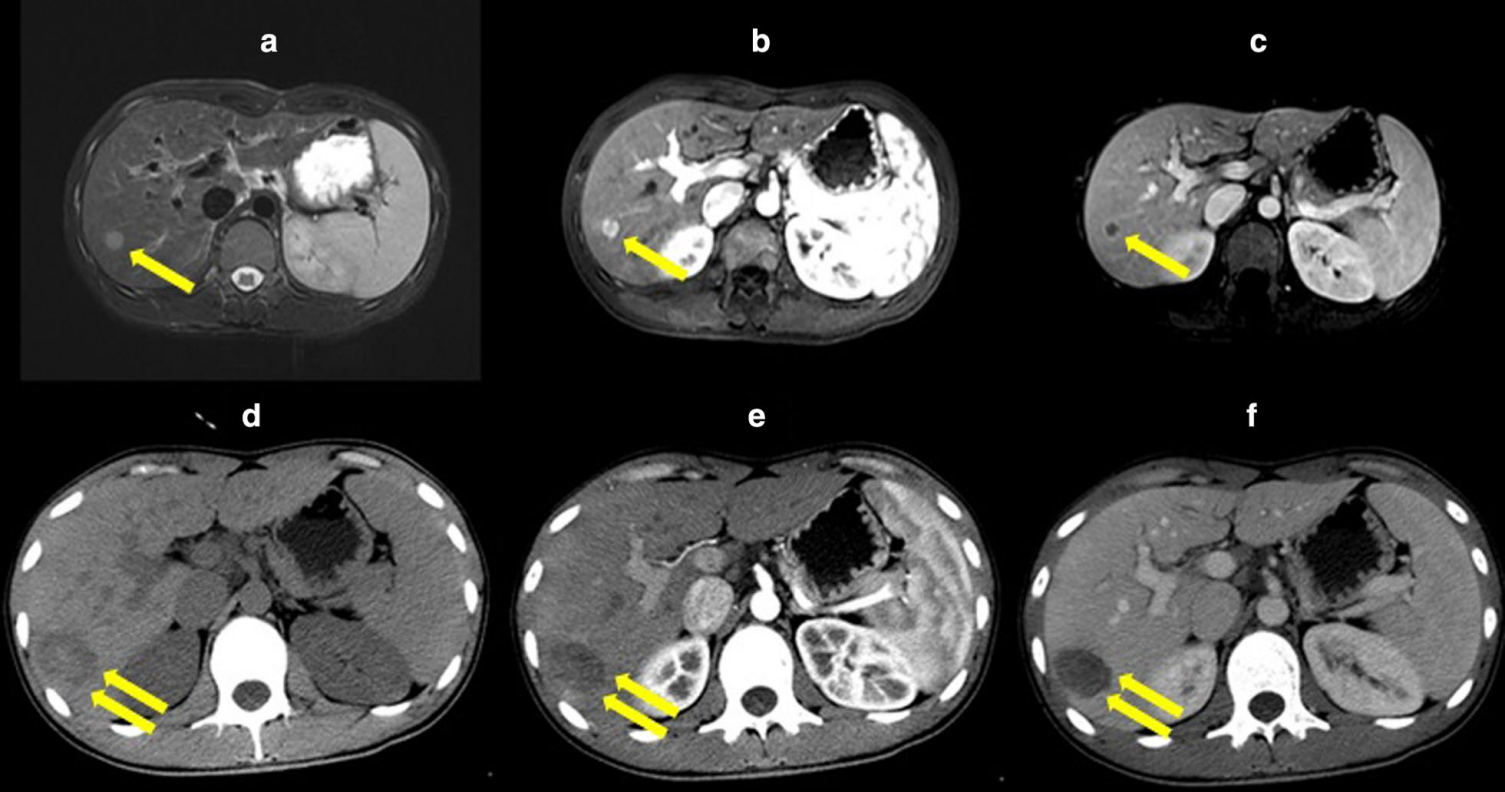
Comparison of pre-treatment and post-treatment tumor lesion images of a 46-year-old patient with hepatocellular carcinoma (HCC) and hepatitis B-related liver cirrhosis who underwent microwave ablation. Magnetic resonance images show a tumor with a small (15-mm) hyperintense nodule (**a** T2-weighted phase), intense arterial enhancement (**b** arterial phase), and enhancement recession (**c** portalvenous phase). Computed tomography images (**d** noncontrast-enhanced phase; **e** arterial phase; **f** portalvenous phase) obtained 2 days after treatment show no contrast enhancement inside or around the ablation zone

A contrast-enhanced CT scan was performed 48 h after ablation. The local efficacy was evaluated according to imaging manifestations [20] and our previous study [9]. Complete ablation was defined as that the ablated area completely covers the target tumor (Fig. 1). Incomplete ablation was defined as any enhancement within the ablation area or the target tumor. All patients with incomplete ablation were further treated by complementary ablations. A major complication was defined as an event that led to substantial morbidity or disability, upgrade of the level of care, or a substantially extended hospital stay [21].

All patients were regularly followed up every 2–3 months during the first 2 years and every 6 months in postoperative 3–5 years. Alpha-fetoprotein (AFP) detection and contrast-enhanced CT/MRI were regularly performed to monitor HCC recurrence. LTP was defined as any new lesion connected to the ablated zone. Distant recurrence (DR) was defined as a new intra-hepatic nodule [21]. Recurrence included LTP and DR. The strategy for managing recurrent HCC was based on simulation, but it was slightly different from the BCLC staging system: (1) for very-early-stage and early-stage HCC with a favorable location for local thermal ablation, repeated ablation was recommended; for very-early-stage and early-stage HCC with an unfavorable location, hepatic resection, if feasible, was recommended; (2) for intermediate- or advanced-stage HCC, transcatheter arterial chemoembolization (TACE) or sorafenib was recommended; (3) for terminal HCC with Child–Pugh A or B cirrhosis, TACE, conservative treatment, or sorafenib was recommended; and (4) for patients with extrahepatic metastasis, systemic chemotherapy was recommended. Treatment for a recurrent tumor was determined by the characteristics of the recurrent tumor and the recommendations of our multidisciplinary team.

The primary endpoint was the 5-year OS rate; the secondary endpoint was RFS rate. Additional points included complete ablation, complication occurrence rates, LTP, and DR. The OS was calculated from the date of ablation to the date of death or last follow-up; the RFS was calculated from the date of ablation to the date of LTP, DR, or last follow-up. The last follow-up date for this study was July 25, 2015.

### Prognostic factor analysis

To identify the prognostic factors for OS, RFS, and LTP, 23 variables were used, including sex, age, etiology, heredity, tumor size, tumor location, Child–Pugh class, hepatitis B virus (HBV)-DNA level, antiviral therapy, alanine aminotransferase, total bilirubin, direct bilirubin (DBIL), albumin (ALB), gamma-glutamyl transpeptidase, PLT, prothrombin time, AFP level, carbohydrate antigen 19-9 (CA19-9), thermal ablation modality, initial local efficiency, LTP, DR, and treatment of recurrence. At the Eastern Hepatobiliary Surgery Hospital, HBV-DNA level of more than 50 copies/mL is considered HBV-DNA positive.

### Statistical analysis

Continuous variables were reported as mean ± standard deviation. Differences in categorical variables and continuous variables between the groups were analyzed with the Chi square test or Fisher’s exact test and with Student’s *t* test, respectively, using the SPSS version 17.0 software (SPSS, Chicago, IL, USA). RFS and OS curves were evaluated using Kaplan–Meier curves and compared using the log-rank test. Variables with *P* values less than 0.05 in the univariate analysis were entered into a Cox proportional hazards model for multivariate analysis. Two-tailed *P* values less than 0.05 were considered statistically significant.

## Results

### Patient groups

A total of 460 patients with HCC were included in this study. Demographic and clinical characteristics of the 460 patients are summarized in Table 1. The follow-up period for this study was at least 3 years. The median follow-up period was 53.0 months (range 8.0–98.0 months) for the 301 patients treated with MWA and 62.0 months (range 6.0–102.0 months) for the 159 patients treated with RFA. No significant differences were observed between the two groups in any preoperative parameters, hospital stay, or follow-up period (*P* = 0.331) (Table 1).

**Table 1:** Demographic and clinical characteristics of 460 patients with hepatocellular carcinoma (HCC)

Parameter	RFA group (*n* = 159)	MWA group (*n* = 301)	*P* value
Sex			0.209
Men	132 (83.0)	235 (78.1)	
Women	27 (17.0)	66 (21.9)	
Age (years)^a^	54.0 ± 11.0	54.2 ± 11.0	0.889
≤65	138 (86.8)	255 (84.7)	0.549
>65	21 (13.2)	46 (15.3)	
Heredity			0.461
Yes	6 (3.8)	16 (5.3)	
No	153 (96.2)	285 (94.7)	
Etiology			0.799
Cryptogenic	21 (13.2)	38 (12.6)	
HBV infection	128 (80.5)	250 (83.0)	
HCV infection	8 (5.0)	11 (3.7)	
Schistosomiasis	2 (1.3)	2 (0.7)	
Tumor size (cm)^a^	1.7 ± 0.3	1.7 ± 0.3	0.335
Tumor location			0.150
Favorable	121 (76.1)	210 (69.8)	
Unfavorable	38 (23.9)	91 (30.2)	
Child–Pugh class			0.127
Class A	140 (88.1)	278 (92.4)	
Class B	19 (11.9)	23 (7.6)	
HBV-DNA^b^			0.076
Positive	70 (44.0)	107 (35.5)	
Negative	58 (36.5)	143 (47.5)	
Absent	31 (19.5)	51 (16.9)	
ALT (U/L)^a^	38.28 ± 23.22	38.37 ± 26.80	0.960
<40	113 (71.1)	237 (78.7)	0.059
40–80	36 (22.6)	57 (18.9)	
>80	10 (6.3)	7 (2.4)	
TBIL (μmol/L)^a^	17.53 ± 8.33	17.51 ± 8.53	0.979
≤20	108 (67.9)	217 (72.1)	0.350
>20	51 (32.1)	84 (27.9)	
DBIL (μmol/L)^a^	6.80 ± 3.91	7.33 ± 4.83	0.236
≤7	104 (65.4)	177 (58.8)	0.167
>7	55 (34.6)	124 (41.2)	
ALB (g/L)^a^	40.60 ± 5.49	41.21 ± 5.31	0.243
<35	25 (15.7)	38 (12.6)	0.358
≥35	134 (84.3)	263 (87.4)	
GGT (U/L)^a^	85.79 ± 83.51	77.98 ± 113.45	0.445
<50	89 (56.0)	187 (62.1)	0.125
50–100	38 (23.9)	75 (24.9)	
>100	32 (20.1)	39 (13.0)	
PLT (×10^9^/L)^a^	120.37 ± 55.13	122.60 ± 60.00	0.696
<100	67 (42.1)	122 (40.5)	0.739
≥100	92 (57.9)	179 (59.5)	
PT (s)^a^	12.9 ± 1.3	12.9 ± 1.3	0.699
≤13	101 (63.5)	185 (61.5)	0.665
>13	58 (36.5)	116 (38.5)	
AFP level (μg/L)			0.144
<20	69 (43.4)	156 (51.8)	
20–200	44 (27.7)	80 (26.6)	
>200	46 (28.9)	65 (21.6)	
CA19-9 (kU/L)			0.053
≤39	107 (67.3)	228 (75.7)	
>39	52 (32.7)	73 (24.3)	
Hospital stay (days)^a^	3.4 ± 1.7	3.4 ± 2.3	0.975

*RFA* radiofrequency ablation, *MWA* microwave ablation, *HBV* hepatitis B virus, *HCV* hepatitis C virus, *ALT* alanine aminotransferase, *TBIL* total bilirubin, *DBIL* direct bilirubin, *ALB* albumin, *GGT* gamma-glutamyl transpeptidase, *PLT* platelet, *PT* prothrombin time, *AFP* alpha fetoprotein, *CA19-9* carbohydrate antigen 19-9

^a^These data are presented as mean ± standard deviation; other values are presented as number of patients followed by percentage in parentheses

^b^HBV-DNA level of more than 50 copies/mL is considered HBV-DNA positive

### Local efficacy

The initial complete ablation rate was 98.3% (296/301) in the MWA group and 98.1% (156/159) in the RFA group, without significant difference (*P* = 0.860). Eight cases of incomplete ablations resulted from tumors with an unfavorable location (Table 2). After complementary MWA (*n* = 5) or RFA (*n* = 3), technical success was achieved in all eight patients.

**Table 2:** Location of tumors with incomplete ablation in 460 HCC patients

Unfavorable location	The entire cohort	RFA group	MWA group
Adjacent to a major vessel	3	1	2
Near pericardium	2	1	1
Near diaphragm	2	1	1
Caudate lobe	1	0	1
Total	8	3	5

*MWA* microwave ablation, *RFA* radiofrequency ablation

### Complications

Ablation-related complications, including pain, fever, and fatigue, were observed in 65.5% (197/301) of patients in the MWA group and 60.4% (96/159) of patients in the RFA group (*P* = 0.282); these symptoms were alleviated after symptom-mitigating treatment. Two patients (0.7%) in the MWA group and one patient (0.6%) in the RFA group experienced major complications. In the MWA group, major complications included intestinal perforation (*n* = 1), which was treated with intestinal surgery, and persistent jaundice (*n* = 1). One patient in the RFA group also experienced persistent jaundice. No significant difference in complication occurrence rates was observed between the two groups (*P* = 0.691). In our study, no ablation-related deaths occurred.

### Recurrence and treatment

During follow-up, HCC recurrence was detected in 52.8% (243/460) of the entire cohort of patients. DR was found in 40.5% (122/301) of patients in the MWA group and 47.8% (76/159) of patients in the RFA group. Twenty-nine patients (9.6%) in the MWA group and 16 patients (10.1%) in the RFA group developed LTP. No significant differences were observed between the RFA and MWA groups in terms of LTP (*P* = 0.883) or DR (*P* = 0.134). Of the 45 cases of LTP, 42 (93.3%) emerged within the first 24 months, and 24 (53.3%) emerged within the first 12 months. At the time of examination, no extrahepatic metastasis was observed. Of the 29 patients in the MWA group with LTP, 27 were treated with repeated MWA, and 4 were treated with hepatic resection. Of the 16 patients in the RFA group with LTP, 14 were treated with repeated RFA, and 2 were treated with hepatic resection. Of the 122 patients in the MWA group diagnosed with DR, 91 were treated with repeated MWA, 18 were treated with TACE, 11 were treated with hepatic resection, and 2 were treated with radiation therapy. Of the 76 patients in the RFA group with DR, 58 were treated with repeated RFA, 9 were treated with TACE, 8 were treated with hepatic resection, and 1 was treated with radiation therapy (Table 3).

**Table 3:** Treatments of HCC patients who developed distant recurrence

Treatment	MWA group (*n* = 122)	REA group (*n* = 76)
Very-early-stage/early-stage HCC		
Repeated ablation	91	58
Hepatic resection	11	8
Intermediate or advanced HCC		
TACE	11	8
Radiation therapy	2	1
Terminal HCC		
TACE	1	1
Sorafenib	0	0

*TACE* transcatheter arterial chemoembolization, *MWA* microwave ablation, *RFA* radiofrequency ablation

### Survival

**Fig. 2:**
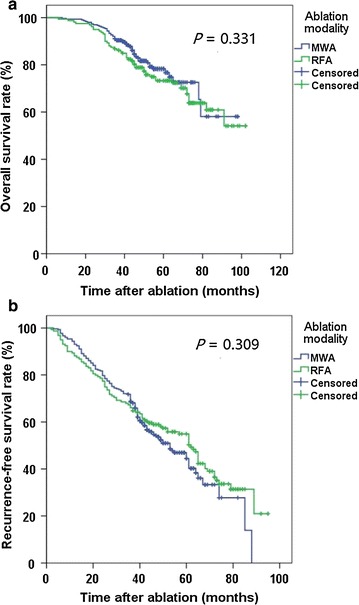
Survival curves for HCC patients treated with radiofrequency ablation (RFA) and microwave ablation (MWA). Survivals were evaluated using Kaplan–Meier curves and compared with the log-rank test. No significant differences are observed between the two groups. **a** Overall survival curves; **b** recurrence-free survival curves

The 1-, 3-, and 5-year OS rates for the MWA group were 99.3%, 90.4%, and 78.3%, respectively, whereas those for the RFA group were 98.7%, 86.8%, and 73.3%, respectively. The 1-, 3-, and 5-year RFS rates for the MWA group were 94.4%, 71.8%, and 46.9%, respectively, whereas those for the RFA group were 89.9%, 67.3%, and 54.9%, respectively. No significant differences were observed between the two groups in OS (*P* = 0.331, Fig. 2a) or RFS (*P* = 0.309, Fig. 2b).

### Prognostic factor analysis for OS and RFS

Univariate analysis showed that antiviral therapy, ALB, AFP level, LTP, and treatment for recurrence were significantly associated with OS. Multivariate analysis showed that antiviral therapy, LTP, and treatment of recurrence were independent prognostic factors (Table 4). In addition, univariate analysis showed that Child–Pugh class, AFP level, and DBIL significantly associated with RFS. Multivariate analysis showed that AFP level was the only independent prognostic factor (Table 5).

**Table 4:** Univariate and multivariate analyses of prognostic factors for overall survival

Variable	Univariate analysis	Multivariate analysis	
	*P* value	Odds ratio (95% CI)	*P* value
Sex: men vs. women	0.222		
Age (years): >65 vs. ≤65	0.762		
Heredity: yes vs. no	0.932		
Etiology: cryptogenic vs. HBV vs. HCV vs. schistosomiasis	0.211		
Tumor location: favorable vs. unfavorable	0.294		
Child–Pugh class: A vs. B	0.083		
HBV-DNA: positive vs. negative vs. absence	0.839		
Antiviral therapy: yes vs. no	0.002	0.468 (0.299–0.734)	0.001
ALT (U/L): <40 vs. 40–80 vs. >80	0.556		
TBIL (μmol/L): ≤20 vs. >20	0.563		
DBIL (μmol/L): ≤7 vs. >7	0.079		
ALB (g/L): <35 vs. ≥35	0.036	1.200 (0.687–2.095)	0.521
GGT (U/L): <50 vs. 50–100 vs. >100	0.366		
PLT (×10^9^/L): <100 vs. ≥100	0.199		
PT (s): ≤13 vs. >13	0.115		
AFP level (μg/L): <20 vs. 20–200 vs. >200	0.014	0.881 (0.679–1.143)	0.341
CA19-9 (kU/L): ≤39 vs. >39	0.878		
TA modality: MWA vs. RFA	0.331		
Initial local efficiency: complete vs. incomplete ablation	0.182		
LTP: presence vs. absence	<0.001	3.711 (2.410–5.714)	<0.001
DR: presence vs. absence	0.152		
Treatment of recurrence: radical vs. palliative	<0.001	0.241 (0.147–0.395)	<0.001

*CI* confidence interval, *HBV* hepatitis B virus, *HCV* hepatitis C virus, *ALT* alanine aminotransferase, *TBIL* total bilirubin, *DBIL* direct bilirubin, *ALB* albumin, *GGT* gamma-glutamyl transpeptidase, *PLT* platelet, *PT* prothrombin time, *AFP* alpha fetoprotein, *TA* thermal ablation, *RFA* radiofrequency ablation, *MWA* microwave ablation, *LTP* local tumor progression, *DR* distant recurrence

**Table 5:** Univariate and multivariate analyses of prognostic factors for recurrence-free survival

Variable	Univariate analysis	Multivariate analysis	
	*P* value	Odds ratio (95% CI)	*P* value
Sex: men vs. women	0.617		
Age (years): >65 vs. ≤65	0.889		
Heredity: yes vs. no	0.553		
Etiology: cryptogenic vs. HBV vs. HCV vs. schistosomiasis	0.414		
Tumor location: favorable vs. unfavorable	0.177		
Child–Pugh class: A vs. B	0.029	0.787 (0.521–1.190)	0.256
HBV-DNA: positive vs. negative vs. absence	0.086		
Antiviral therapy: yes vs. no	0.179		
ALT (U/L): <40 vs. 40–80 vs. >80	0.117		
TBIL (μmol/L): ≤20 vs. >20	0.117		
DBIL (μmol/L): ≤7 vs. >7	0.022	1.237 (0.939–1.629)	0.130
ALB (g/L): <35 vs. ≥35	0.105		
GGT (U/L): <50 vs. 50–100 vs. >100	0.268		
PLT (×10^9^/L): <100 vs. ≥100	0.073		
PT (s): ≤13 vs. >13	0.157		
AFP level (μg/L): <20 vs. 20–200 vs. >200	<0.001	1.370 (1.178–1.593)	<0.001
CA19-9 (kU/L): ≤39 vs. >39	0.772		
TA modality: MWA vs. RFA	0.309		
Initial local efficiency: complete vs. incomplete ablation	0.429		

*CI* confidence interval, *HBV* hepatitis B virus, *HCV* hepatitis C virus, *ALT* alanine aminotransferase, *TBIL* total bilirubin, *DBIL* direct bilirubin, *ALB* albumin, *GGT* gamma-glutamyl transpeptidase, *PLT* platelet, *PT* prothrombin time, *AFP* alpha fetoprotein, *TA* thermal ablation, *RFA* radiofrequency ablation, *MWA* microwave ablation

### Subgroup survival

**Fig. 3:**
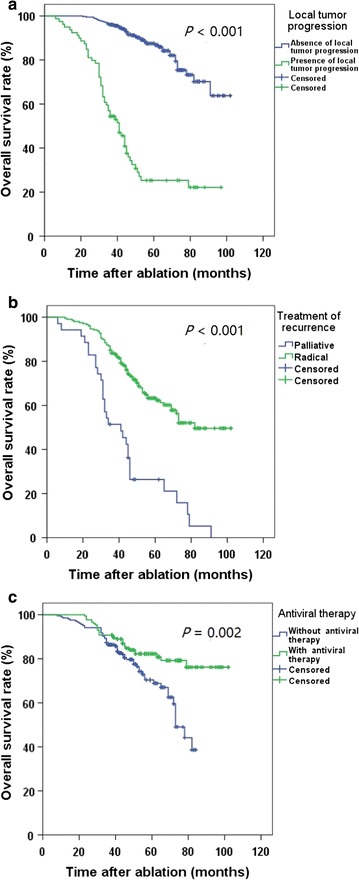
Overall survival curves for HCC patients in different subgroups. Survivals were evaluated using Kaplan–Meier curves and compared with the log-rank test. **a** The overall survival rates of patients without local tumor progression (LTP) are higher than those of patients with LTP (*P* < 0.001). **b** The overall survival rates of patients who received radical therapies are higher than those of patients who received palliative therapies (*P* < 0.001). **c** The overall survival rates of patients who received antiviral therapy are higher than those of patients who did not receive antiviral therapy (*P* = 0.002)

For patients without LTP, the 1-, 3-, and 5-year OS rates were 100%, 96.3%, and 87.5%, respectively, which were significantly higher than those in patients with LTP (98.7%, 57.0%, and 25.3%, respectively; *P* < 0.001) (Fig. 3a). For the treatment of recurrence, the 1-, 3-, and 5-year OS rates were 99.0%, 85.1%, and 63.3%, respectively, in patients who received radical treatments, including repeated ablation and hepatic resection, which were significantly higher than those in patients who received palliative treatments, including TACE and radiation therapy (94.3%, 51.4%, and 26.4%, respectively; *P* < 0.001) (Fig. 3b). Also, the 1-, 3-, and 5-year OS rates in HBV patients who received antiviral therapy (100.0%, 90.8%, and 82.2%, respectively) were significantly higher than those in patients who did not receive antiviral therapy (98.5%, 87.3% and 70.4%, respectively; *P* = 0.002) (Fig. 3c).

## Discussion

We found no statistically significant differences in the rates of complete ablation, LTP, and procedure-related major complications between patients who underwent MWA and patients who underwent RFA; we also found no statistically significant differences in 5-year RFS and OS rates. Patients who did not receive antiviral therapy or who received palliative treatment therapy had short OS, and LTP occurrence was a predictor for short OS. Patients with a high AFP level tended to have a high LTP rate and short RFS.

The initial complete ablation rates were 98.3% in the MWA group and 98.1% in the RFA group, which were similar to those reported in previous studies [11–13]. Theoretically according to our previous study, MWA could generate a wider necrosis zone [9]. Since the size of very-early-stage HCC in this study was no larger than 2 cm, a sufficient necrotic zone was created by both MWA and RFA, and no significant difference was observed. In recognizing the similar complete ablation efficacy, no recommendation was provided to patients to select one modality over the other. We only recommended MWA for tumors that were adjacent to major vessels to reduce a potential heat sink effect. Having some nodules located in high layers near the pericardium diaphragm, for instance, represents an unfavorable location, and this poses difficulty for probing procedures. In our experience, a proper lowering of the puncture site could allow the antenna probe to be inserted deeper into the liver tissue, leading to better ablation efficacy. A low complication rate is a notable advantage of thermal ablation [22, 23]. In this cohort, only three patients had major complications. The complication rates were 0.7% and 0.6% for MWA and RFA, respectively. Thus, in treating very-early-stage HCC, MWA was as safe as RFA.

For HCC of 2 cm or smaller, the 5-year LTP rates were reported to be 10%–15.9% after RFA [11, 17, 24]. In our study, no significant difference was observed between the two groups in the LTP rate: 9.6% in the MWA group and 10.1% in the RFA group. LTP is mainly associated with tumor size [24], which was within 2 cm in both groups, thus explaining why the occurrence rates of LTP were similar.

Although the selection of thermal ablation as the first treatment option is not universal, most studies have shown a satisfactory prognosis after thermal ablation treatments. Roayaie et al. [14] analyzed the prognostic data of 132 patients with HCC of 2 cm or smaller who underwent hepatic resection at two Western centers; they found that, after hepatic resection, the median survival was 74.5 months and the 5-year survival rate was 70%. In a retrospective study of databases in five hospital departments, Livraghi et al. [12] found that, after treatment with RFA, the 5-year survival rate was 68.5%. Similarly, Peng et al. [13] retrospectively compared RFA with hepatic resection in 145 patients with HCC of 2 cm or smaller. They found that the 5-year OS rate was 71.9% with RFA and 62.1% with hepatic resection; moreover, the corresponding RFS rates were 59.8% with RFA and 51.3% with hepatic resection. In our study, no significant difference was observed between the two groups in the 5-year OS rate: 78.3% for the MWA group and 73.3% for the RFA group. Our 5-year OS rates were approximately the same as those found in previous studies [11, 12]; importantly, they were similar to the outcomes of patients treated with hepatic resection [14]. The 5-year RFS rates were 46.9% for the MWA group and 54.9% for the RFA group (*P* > 0.05), which were consistent with the findings of Kuang et al. [11] but higher than those reported in other studies [12, 15–17]. A primary reason for the discrepancy was that, in those studies, RFA or MWA was selected for HCC patients who were unsuitable for hepatic resection because of severe liver function impairment or deteriorated underlying conditions. In contrast, in the present study, thermal ablation was the first-choice treatment for very-early-stage HCC patients, the majority of whom had resectable HCC.

Notably, in the subgroup analysis, the OS of patients with LTP was much shorter than that of patients with DR. Most patients (42/45, 93.3%) had LTP within the first 24 months. Of the 45 patients with LTP, 24 (53.3%) were found within the first 12 months. A short RFS is a significant risk factor that compromised the OS of HCC patients who were treated with hepatic resection or RFA [25, 26]. The emergence of LTP significantly shortened RFS and served as a predictor of poor prognosis.

Many studies have suggested that high AFP level is an unfavorable factor for RFS [6, 7, 27, 28]. Higher AFP level may be associated with more severe cirrhosis, more frequent vascular invasion, higher tumor burden, and poorer prognosis [27]. Our results yielded similar conclusions.

High HBV-DNA level has been reported to associate with high postoperative recurrence, which has a negative effect on postoperative survival [29]. Antiviral therapy inhibits HBV replication, fibrosis, and carcinogenesis. Indeed, in our study, antiviral therapy was found to be a favorable prognostic factor for OS, as shown by univariate and multivariate analyses. In 40%–70% of patients, intrahepatic HCC recurred within 5 years after the primary treatments [30, 31]. In a recent study, 52.8% of patients experienced HCC recurrence after the first ablation. The strategy for managing recurrent HCC was based on a simulation that was slightly different from the BCLC staging system. We observed that patients who received radical therapies, including hepatic resection and repeated ablation, achieved longer OS than those who received palliative therapies. There are three possible reasons for this observation. First, radical therapies were recommended to patients with a lighter tumor burden, which primarily determined the prognosis. Second, the efficiency of palliative therapies is limited and barely necrotizes nodules. Third, palliative therapies are usually indicated for patients with recurrent HCC who have impaired liver function, which results in a poor prognosis.

The Eastern Hepatobiliary Surgery Hospital is the largest hepatobiliary surgery center in Asia; as such, we handle the largest number of HCC ablations every year. To our knowledge, this study presents the largest number of patients with very-early-stage HCC, as well as the superlative comparison of efficacy and safety between MWA and RFA in treating this disease. Our study did have several limitations. First, this was a single-institution, retrospective, comparative study. Second, as a retrospective study, a subjective selection bias was inherently embedded. Third, no explicit guidelines determined which patients should be referred to RFA or MWA treatment. Future prospective, randomized controlled trials are needed to confirm these findings.

Although MWA does have some advantages over RFA, our results showed that both modalities achieved similar OS and RFS when the rate of LTP was similar. Therefore, despite RFA being the current leading option for the nonsurgical treatment of HCC, MWA warrants more attention and should be given preferable consideration when selecting therapy for patients with very-early-stage HCC.

## Conclusions

In summary, our findings suggest that, as assessed by OS, RFS, complete ablation, LTP, DR, and complications, MWA is as safe and effective as RFA in treating very-early-stage HCC. Both MWA and RFA should be considered first treatment options for very-early-stage HCC.

## Abbreviations

HCC: hepatocellular carcinoma
RFA: radiofrequency ablation
MWA: microwave ablation
LTP: local tumor progression
OS: overall survival
RFS: recurrence-free survival
DR: distant recurrence
